# Syntactic complexity and diversity of spontaneous speech production in schizophrenia spectrum and major depressive disorders

**DOI:** 10.1038/s41537-023-00359-8

**Published:** 2023-05-29

**Authors:** Katharina Schneider, Katrin Leinweber, Hamidreza Jamalabadi, Lea Teutenberg, Katharina Brosch, Julia-Katharina Pfarr, Florian Thomas-Odenthal, Paula Usemann, Adrian Wroblewski, Benjamin Straube, Nina Alexander, Igor Nenadić, Andreas Jansen, Axel Krug, Udo Dannlowski, Tilo Kircher, Arne Nagels, Frederike Stein

**Affiliations:** 1 Department of English and Linguistics, General Linguistics, University of Mainz, Mainz, Germany; 2 Department of Psychiatry and Psychotherapy, University of Marburg, Marburg, Germany; 3 Center for Mind, Brain and Behavior, University of Marburg, Marburg, Germany; 4 Department of Psychiatry and Psychotherapy, University of Bonn, Bonn, Germany; 5 Institute for Translational Psychiatry, University of Münster, Münster, Germany

**Keywords:** Schizophrenia, Psychosis

## Abstract

Syntax, the grammatical structure of sentences, is a fundamental aspect of language. It remains debated whether reduced syntactic complexity is unique to schizophrenia spectrum disorder (SSD) or whether it is also present in major depressive disorder (MDD). Furthermore, the association of syntax (including syntactic complexity and diversity) with language-related neuropsychology and psychopathological symptoms across disorders remains unclear. Thirty-four SSD patients and thirty-eight MDD patients diagnosed according to DSM-IV-TR as well as forty healthy controls (HC) were included and tasked with describing four pictures from the Thematic Apperception Test. We analyzed the produced speech regarding its syntax delineating measures for syntactic complexity (the total number of main clauses embedding subordinate clauses) and diversity (number of different types of complex sentences). We performed cluster analysis to identify clusters based on syntax and investigated associations of syntactic, to language-related neuropsychological (verbal fluency and verbal episodic memory), and psychopathological measures (positive and negative formal thought disorder) using network analyses. Syntax in SSD was significantly reduced in comparison to MDD and HC, whereas the comparison of HC and MDD revealed no significant differences. No associations were present between speech measures and current medication, duration and severity of illness, age or sex; the single association accounted for was education. A cluster analysis resulted in four clusters with different degrees of syntax across diagnoses. Subjects with less syntax exhibited pronounced positive and negative symptoms and displayed poorer performance in executive functioning, global functioning, and verbal episodic memory. All cluster-based networks indicated varying degrees of domain-specific and cross-domain connections. Measures of syntactic complexity were closely related while syntactic diversity appeared to be a separate node outside of the syntactic network. Cross-domain associations were more salient in more complex syntactic production.

## Introduction

Given the limitations of current psychiatric classification, a number of studies have attempted to disentangle the heterogeneity and comorbidity across affective and psychotic disorders (i.e., major depressive disorder (MDD), schizophrenia spectrum disorder (SSD), bipolar disorder (BD)) using transdiagnostic and multivariate approaches including symptomology, neuroimaging, and blood-specimen measures^1–7^. However, studies have failed to identify reproducible biomarkers for the aforementioned psychiatric disorders^8–10^. Recent studies have highlighted the importance of speech features as speech aberrations have a high prognostic value for onset, course, chronicity, and treatment response of SSD as well as MDD^11^. Hereof, speech is considered to be an objective and specifically quantitative measure for obtaining and analyzing that is reproducible, time efficient and non-invasive in nature^12,13^.

A few typical language-related symptoms of SSD include reduced speech production^14,15^ and reduced performances in verbal fluency tasks^16,17^ along with difficulty in word-retrieval which lead to word approximations^18^, production of neologisms^19,20^, and less complexity of sentences^14,15,21–25^. These linguistic aberrations can even be observed in early stages prior to manifestation of the disorder^25–27^. In MDD, speech is mainly characterized by longer response latencies and reduced spontaneous speech^13^. Additionally, depressive speech often contains a higher rate of first-person singular pronouns and self-focused language, which is characterized by words related to sad emotions and the past^28,29^. A greater amount of more truncated and more impersonal sentences was detected on the syntactic level in comparison to healthy controls (HC)^28^. These alterations in multiple domains of speech can be summarized as the qualitative rating of formal thought disorder (FTD)^30^. FTD is not an unique symptom of SSD, but occurs in other psychiatric disorders such as MDD^31–33^. While there is much evidence about reduced syntactic complexity in SSD, it remains unknown how individuals with SSD differ from HC and those with MDD concerning the use of different types of subordinate clauses^30,33^.

The number of produced simple sentences reveals no differences between SSD and HC^34^. The simplest syntactic version on a sentence level is a main clause without embedded sentences, whereas complex sentences consist of multiple merged clauses^35^. Complex sentences can be considered coordinated structures which contain independent parts of sentences connected by a conjunction, and subordinate structures, which consist of a main clause and at least one subordinate clause that depends on the main clause^35^. These subordinate clauses are often underrepresented in the speech production in SSD^14,15,21–24^, however, the scope of complex sentences amongst studies is varied.

A recent increase in use of natural language processing (NLP) measures were shown to distinguish SSD patients from HC with accuracies between 70–94%^36–39^. A multitude on various aspects of language, e.g., phonetic features, coherence, structure in written or spoken language, have already been investigated by NLP^12^. In the present study, we focused on syntax (i.e., complexity and diversity) in language analysis due to its evidence-based connection to cognitive variables such as executive functioning and working memory in SSD^14,18^. Thus, findings on syntax can provide insight into the underlying cognitive processes involved in speech production^40^. The importance of syntactic complexity for diagnosis and monitoring of SSD has been previously demonstrated in several studies^25,40–42^. In addition, syntax can be useful for gaining deeper insight into the nature and severity of language and communication impairments in SSD^40,42^. In contrast to other NLP measures, e.g., prosodic features or idea density^12^, the use of subordinate clauses enables speakers to convey coherent information and especially reflect on complex ideas in discourses^43^. Therefore, a reduced complexity of speech leads to a restricted expression of thoughts during social communication^25^. Listeners may show greater difficulties in drawing conclusions due to a lack of syntactic organization by subordination accompanied by questions or misunderstandings^44,45^. As a result, speakers are required to provide additional information and may feel frustrated about the inability to have a smooth conversation. However, there is a lack of evidence of the production of different types of subordinate clauses in German transdiagnostic samples. Our intention, therefore, was to broaden the view on syntax of spoken language by including MDD in this analysis. An overlap in psychopathology amongst psychotic and affective disorders is well known, thus we intended to expand the knowledge on a language domain^30,33^.

Based on previous studies investigating syntax, the following questions remain unanswered: (1) What is the frequency of subordinate clauses which includes all types of adverbial clauses, relative clauses, complement clauses, and indirect questions in German oral language production in HC, MDD, and SSD?, (2) How do individuals with SSD differ in producing subordinate clauses from HC and those with MDD?, (3) Do participants with a lower syntactic complexity and diversity differ in terms of language-related neuropsychology and psychopathology from those with higher syntactic performance regardless of psychiatric diagnosis? (4) What kind of sub-networks consisting of syntactic measures, language-related neuropsychology, and psychopathology can be detected in relation to the degree of syntax? To address these questions, a classification algorithm was used to examine the possibility of differentiation of syntactic complexity and diversity between SSD, MDD, HC in our sample. Furthermore, we wanted to shed light on networks of syntax, language-related neuropsychology, and psychopathology in all participants. Hereof, we hypothesized that patients suffering from SSD are less likely to produce complex speech when compared to HC^14,15,21–24^ while those with MDD are comparable to HC. Language scores indicate a significant difference in relation to their distribution in SSD when compared to MDD^46^, and MDD show less symptoms of poverty of speech than SSD^47^. Moreover, we anticipated a negative relationship between syntax and negative^48^ and positive symptoms^14^.

## Results

SSD yielded significantly less semantic verbal fluency (VF), alternating VF, and verbal episodic memory than MDD and HC. Moreover, SSD showed significantly more negative and positive symptoms in all subscales of the Scale for Assessment of Negative Symptoms (SANS) and Positive Symptoms (SAPS) in comparison to MDD and HC. Exclusively, the total sum of SANS indicated significant differences between MDD and HC. For more details see Table 1.

**Table 1:** Descriptive data of participants.

	Schizophrenia spectrum disorders (*n* = 34)	Major depressive disorder (*n* = 38)	Healthy controls (*n* = 40)	Group comparison	Effect size
Descriptives					
Age	42.47 (13.11)	40.82 (13.03)	40.83 (13.35)	*p* = 0.791 (F = 0.347)	*η*^*2*^ = 0.003
Sex	f = 10 m = 24	f = 26 m = 12	f = 25 m = 15	***p*** = **0**.**002**^**a**^ (χ^2^ = 12.63)	*V* = 0.336
IQ	109.28 (14.57)	109.84 (15.46)	115.95 (15.89)	*p* = 0.139 (H = 3.95)	*η*^*2*^ = 0.039
Education	12.00 (2.32)	13.11 (2.56)	14.79 (2.65)	***p*** < **0**.**001**^**b**^ (F = 10.79)	*η*^*2*^ = 0.173
Medication					
Antidepressants (%)	6 (17.65)	14 (36.84)	–	***p*** < **0**.**001**^**c**^ (χ^2^ = 18.03)	*V* = 0.214
Antipsychotics (%)	21 (61.76)	2 (5.26)	–	***p*** < **0**.**001**^**d**^ (χ^2^ = 51.19)	*V* = 0.605
Mood stabilizers (%)	6 (17.65)	1 (2.63)	–	***p*** < **0**.**001**^**d**^ (χ^2^ = 4.67)	*V* = 0.218
Medication load index	1.78 (1.77)	0.53 (0.75)	–	***p*** < **0**.**001**^**d**^ (F = 23.29)	*η*^*2*^ = 0.303
Sackeim total score	0.47 (1.11)	0.97 (1.4)	–	***p*** < **0**.**001**^**c**^ (F = 8.48)	*η*^*2*^ = 0.143
chlorpromazine equivalents total score	402.54 (773.65)	0.55 (3.21)	–	***p*** < **0**.**001**^**d**^ (F = 10.35)	*η*^*2*^ = 0.218
Duration and severity of illness					
Number of hospitalizations	3.03 (2.90)	1.39 (2.15)	–	***p*** = **0**.**008**^**d**^ (F = 7.41)	*η*^*2*^ = 0.097
Duration of hospitalization	23.37 (26.19)	7.20 (10.24)	–	***p*** < **0**.**001**^**d**^ (F = 12.34)	*η*^*2*^ = 0.152
Duration of current episode	52.73 (87.06)	19.92 (26.41)	–	*p* = 0.138 (F = 2.32)	*η*^*2*^ = 0.069
Neuropsychology					
Semantic VF	18.94 (5.45)	23.47 (5.43)	22.51 (4.39)	***p*** = **0**.**001**^**e**^ (F = 7.39)	*η*^*2*^ = 0.127
Phonemic VF	10.00 (4.46)	11.18 (4.24)	10.66 (3.93)	*p* = 0.505 (F = 0.688)	*η*^*2*^ = 0.013
Alternating VF	11.63 (3.75)	14.74 (3.10)	15.63 (2.41)	***p*** < **0**.**001**^**e**^ (H = 21.91)	*η*^*2*^ = 0.229
Verbal episodic memory^h^	46.16 (8.23)	59.58 (8.20)	58.94 (9.72)	***p*** < **0**.**001**^**e**^ (F = 25.11)	*η*^*2*^ = 0.328
Psychopathology					
SANS sum	17.11 (12.77)	5.12 (6.67)	1.38 (5.12)	***p*** < **0**.**001** ^**f**^ (H = 54.57)	*η*^*2*^ = 0.376
SANS affect	5.93 (5.01)	1.32 (2.97)	0.63 (2.30)	***p*** < **0**.**001** ^**g**^ (H = 35.79)	*η*^*2*^ = 0.304
SANS alogia^i^	2.55 (2.65)	0.66 (1.07)	0.16 (0.53)	***p*** < **0**.**001** ^**g**^ (H = 33.93)	*η*^*2*^ = 0.286
SANS avolition	5.07 (3.91)	1.37 (1.95)	0.18 (0.67)	***p*** < **0**.**001** ^**f**^ (H = 47.51)	*η*^*2*^ = 0.414
SANS anhedonia	4.02 (4.65)	1.69 (3.19)	0.38 (1.94)	***p*** < **0**.**001** ^**g**^ (H = 28.08)	*η*^*2*^ = 0.166
SAPS sum	14.25 (14.11)	1.28 (2.58)	0.57 (2.03)	***p*** < **0**.**001** ^**g**^ (H = 43.59)	*η*^*2*^ = 0.377
SAPS hallucinations	2.69 (4.95)	0.05 (0.23)	0.00 (0.00)	***p*** < **0**.**001** ^**g**^ (H = 28.71)	*η*^*2*^ = 0.172
SAPS delusions	4.52 (5.82)	0.29 (1.78)	0.23 (1.14)	***p*** < **0**.**001** ^**g**^ (H = 42.99)	*η*^*2*^ = 0.250
SAPS bizarre behaviour	0.70 (0.97)	0.03 (0.16)	0.05 (0.32)	***p*** < **0**.**001** ^**g**^ (H = 28.55)	*η*^*2*^ = 0.222
SAPS positive FTD	7.09 (8.19)	1.08 (1.96)	0.28 (0.96)	***p*** < **0**.**001** ^**g**^ (H = 33.88)	*η*^*2*^ = 0.292

Means and standard deviations (SD) (in brackets) are listed for each group and category. Pairwise comparisons:

^a^ = significant difference between SSD and MDD, HC

^b^ = SSD, MDD < HC.

^c^ = SSD < MDD.

^d^ = SSD > MDD.

^e^ = SSD < MDD, HC.

^f^ = SSD > MDD > HC.

^g^ = SSD > MDD, HC.

^h^Sum of correct words.

^i^Subscale used for negative FTD.

Bold font indicates significant results after correcting for multiple testing (Bonferroni).

### Linguistic parameters

We used ANOVA analyses to investigate differences in syntactic speech production between SSD, MDD, and HC. The group differences of the syntactic complexity, diversity and the sub-categories of complex sentences are presented in Table 2. Furthermore, we tested whether syntactic complexity and diversity were associated with possible confounders using a correlation analysis. The extracted measures of syntactic complexity and diversity did not correlate to current medication (chlorpromazine equivalents^49^, Sackeim score^50^, medication load index)^51^, duration and severity of illness (number of hospitalizations, duration of hospitalization, and duration of current episode), age or sex (all *p*s > 0.05), but syntactic diversity correlated with years of education (*r* = 0.33, *p* < 0.001) (see Extended Data Table 1).

**Table 2:** Analyzed linguistic parameters.

	Schizophrenia spectrum disorders (*n* = 34)	Major depressive disorder (*n* = 38)	Healthy controls (*n* = 40)	Group comparison	Effect size
Total number of words	1023.47 (428.4)	1157.76 (333.6)	1148.25 (306.82)	*p* = 0.213 (F = 1.57)	*η*^*2*^ = 0.028
Total number of sentences	77.0 (35.34)	63.58 (19.83)	65.35 (16.68)	*p* = 0.492 (H = 1.42)	*η*^*2*^ = 0.054
MLU	13.8 (3.74)	18.76 (4.74)	17.91 (4.18)	***p*** < **0**.**001**^**a**^ (F = 13.80)	*η*^*2*^ = 0.202
Total number of different words	320.79 (108.61)	372.42 (80.77)	370.95 (90.55)	*p* = 0.033^a^ (F = 3.52)	*η*^*2*^ = 0.061
TTR	0.33 (0.05)	0.33 (0.05)	0.33 (0.04)	*p* = 0.871 (F = 0.14)	*η*^*2*^ = 0.003
Simple sentences^e^	0.35 (0.09)	0.22 (0.08)	0.23 (0.08)	***p*** < **0**.**001**^**b**^ (F = 24.05)	*η*^*2*^ = 0.306
Coordinated sentences^e^	0.48 (0.13)	0.63 (0.12)	0.63 (0.10)	***p*** < **0**.**001**^**a**^ (F = 19.69)	*η*^*2*^ = 0.265
Relative sum of subordinate clauses	0.33 (0.11)	0.43 (0.13)	0.41 (0.12)	***p*** < **0**.**001**^**a**^ (F = 7.4)	*η*^*2*^ = 0.120
Extended relative sum of subordinate clauses	0.48 (0.23)	0.71 (0.33)	0.68 (0.28)	***p*** = **0**.**002**^**a**^ (F = 6.64)	*η*^*2*^ = 0.109
Pure syntactic complexity	1.43 (0.26)	1.62 (0.37)	1.64 (0.28)	***p*** = **0**.**008**^**a**^ (F = 5.06)	*η*^*2*^ = 0.085
Weighted sum of subordinate clauses	0.74 (0.45)	1.26 (0.91)	1.21 (0.65)	***p*** = **0**.**003**^**a**^ (F = 6.05)	*η*^*2*^ = 0.100
Syntactic diversity	0.52 (0.13)	0.62 (0.13)	0.63 (0.14)	***p*** = **0**.**002**^**a**^ (F = 6.81)	*η*^*2*^ = 0.111
Relative clauses^e^	0.09 (0.07)	0.13 (0.08)	0.14 (0.08)	*p* = 0.017^c^ (F = 4.24)	*η*^*2*^ = 0.072
Temporal clauses^e^	0.00 (0.01)	0.01 (0.01)	0.00 (0.01)	*p* = 0.083 (F = 2.55)	*η*^*2*^ = 0.045
Local clauses^e^	0.01 (0.02)	0.01 (0.01)	0.01 (0.01)	*p* = 0.924 (F = 0.08)	*η*^*2*^ = 0.001
Modal clauses^e^	0.00 (0.00)	0.01 (0.01)	0.01 (0.01)	*p* = 0.039^c^ (H = 6.50)	*η*^*2*^ = 0.054
Causal clauses^e^	0.05 (0.04)	0.07 (0.06)	0.07 (0.05)	*p* = 0.279 (F = 1.29)	*η*^*2*^ = 0.023
Conditional clauses^e^	0.02 (0.03)	0.03 (0.03)	0.03 (0.03)	*p* = 0.228 (F = 1.5)	*η*^*2*^ = 0.027
Adversative clauses^e^	0.00 (0.01)	0.00 (0.01)	0.00 (0.01)	*p* = 0.315 (F = 1.17)	*η*^*2*^ = 0.021
Final clauses^e^	0.01 (0.01)	0.02 (0.02)	0.02 (0.02)	*p* = 0.034^c^ (F = 3.5)	*η*^*2*^ = 0.060
Consecutive clauses^e^	0.01 (0.01)	0.02 (0.04)	0.01 (0.02)	*p* = 0.072 (F = 2.69)	*η*^*2*^ = 0.047
Concessive clauses^e^	0.00 (0.01)	0.01 (0.02)	0.01 (0.01)	*p* = 0.289 (F = 1.26)	*η*^*2*^ = 0.023
Comparative clauses^e^	0.05 (0.04)	0.07 (0.04)	0.07 (0.05)	*p* = 0.13 (F = 2.08)	*η*^*2*^ = 0.037
Complement clauses^e^	0.11 (0.07)	0.15 (0.09)	0.15 (0.07)	*p* = 0.038^d^ (F = 3.37)	*η*^*2*^ = 0.058
Indirect questions^e^	0.06 (0.04)	0.07 (0.05)	0.06 (0.03)	*p* = 0.261 (F = 1.36)	*η*^*2*^ = 0.024

Means and standard deviations (SD) (in brackets) are listed for each group and category. Pairwise comparisons:

^a^ = SSD < MDD, HC.

^b^ = SSD > MDD, HC.

^c^ = SSD < HC.

^d^ = SSD < MDD.

^e^Values are in relation to the total number of sentences.

Bold font indicates significant results after correcting for multiple testing (Bonferroni).

### Classification

We used classification analyses to investigate the diagnostic utility of syntactic complexity and diversity. Classification accuracies for HC vs SSD, HC vs MDD, and SSD vs MDD were 0.66 (*p* < 0.004), 0.51 (*p* < 0.35), and 0.63 (*p* < 0.005).

### Cluster analysis

Cluster analysis was used to investigate transdiagnostic clusters underlying syntactic measures. Four clusters with a Bayesian Information Criterion (BIC) of 294.88 could be shown (cluster 1: *n* = 39, cluster 2: *n* = 19, cluster 3: *n* = 20, cluster 4: *n* = 34) ranging from extremely complex to very, moderate, and slightly complex speech. Out of the total number of participants in the extremely complex cluster, 45% were HC, another 45% had MDD, and only 10% had been diagnosed with SSD. An inverse distribution of diagnoses was indicated in the slightly complex cluster: 58.8% SSD, 23.5% MDD and 17.6% HC. Nevertheless, all clusters contained participants of SSD, MDD and HC, indicating a transdiagnostic distribution. Interaction analyses were used to test if the distribution of participants to one of the four clusters was driven by clinical diagnoses. No interaction effect was found for any of the five measures representing syntactic complexity and diversity (all *p*s > 0.05). The identified clusters did not differ in current medication intake (chlorpromazine equivalents^49^, Sackeim score^50^, medication load index)^51^, duration and severity of illness (hospitalizations, duration of hospitalization, and duration of current episode), age or sex, but the extremely complex cluster differed from the slightly complex cluster in relation to years of education (*p* = 0.005) (see Extended Data Table 2). The results of one-way ANOVAs or Kruskal-Wallis tests to compare language-related neuropsychological and psychopathological data of these four groups based on clustering of syntax are listed in Extended Data Table 3.

### Network analyses

**Fig. 1:**
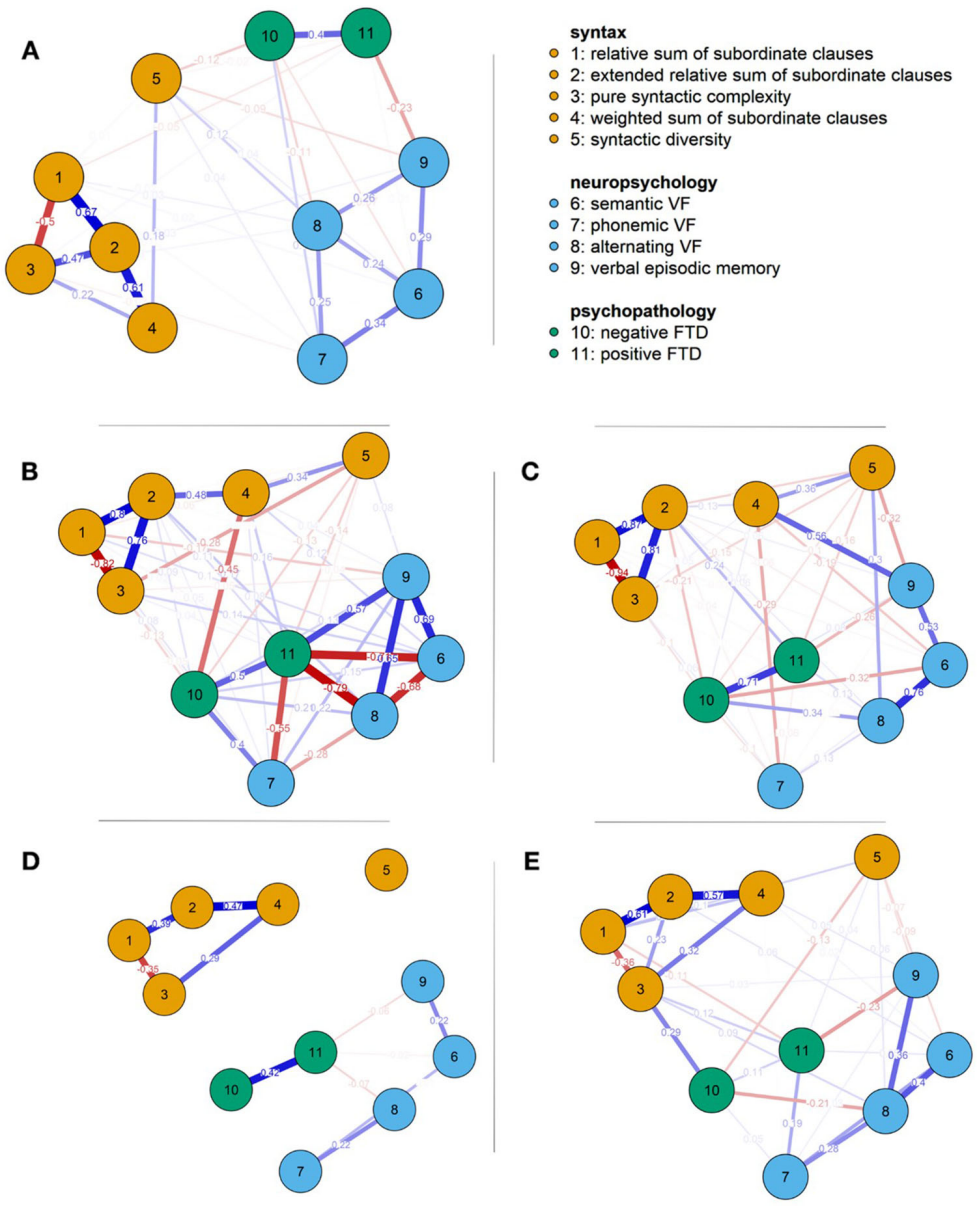
Networks over all participants and in clusters. Networks using the EBICglasso method over all participants (**A**) and in clusters: extremely complex cluster (**B**), very complex cluster (**C**), moderately complex (**D**), slightly complex (**E**) based on Gaussian Graphical Model including variables of syntax, neuropsychology, and psychopathology. All correlations illustrated in the networks as edges are regularized partial correlations and stronger than 0.1 and −0.1. Estimated correlations with the value 0 are not visualized in the network. Orange nodes are part of syntax represented by (1) relative sum of subordinate clauses; (2) extended relative sum of subordinate clauses; (3) pure syntactic complexity; (4) weighted sum of subordinate clauses; (5) syntactic diversity, blue nodes to neuropsychology represented by (6) semantic VF; (7) lexical VF; (8) alternating VF; (9) verbal episodic memory, and green nodes to psychopathology represented by (10) negative FTD and (11) positive FTD. Blue connections indicate positive relationships, red connections mark negative relationships. The thickness of lines represents the weight of connections.

The network of the full sample presented in Fig. 1A was characterized by associations within each domain (syntactic complexity, language-related neuropsychology, and psychopathology) in the range of weak to strong correlations whereas cross-domain connections were very weak. The extended relative sum of subordinate clauses appeared with highest expected influence (EI) and strength (S) (EI = 2.29; S = 2.25) in all participants. Strength measures were followed by relative sum of subordinate clauses (S = 0.87) and pure syntactic complexity (S = 0.85) (see Extended Data Table 3, Extended Data Fig. 1). The four cluster-based networks in Fig. 1B–E illustrated more impactful links between the domains; thus, sub-networks were intercorrelated in all clusters to a different extent. All networks are shown in Fig. 1 and centrality measures are listed in Extended Data Table 4 and plotted in Extended Data Fig. 1.

In summary, the extended relative sum of subordinate clauses indicated a very relevant node in all networks. Furthermore, both syntactic complexity and diversity were associated with positive and negative FTD except in the moderately complex cluster. Finally, it appeared that associations between syntax, language-related neuropsychology, and psychopathology were more pronounced in more complex syntactic production (i.e., the extremely complex cluster) than in participants with reduced syntactic performance (i.e., the slightly complex and moderately complex cluster). See Extended Data Fig. 2 for an insight into the networks of HC, SSD, and MDD.

## Discussion

The aim of the current study was to analyze syntax in oral speech production in individuals with SSD compared to HC and those with MDD. Moreover, we investigated networks based on a subset of syntactic, language-related neuropsychological, and psychopathological measures. Results indicated significantly higher syntax in HC and MDD when compared to those with SSD. Classification analyses revealed significant results supporting this finding, albeit with poor performance. Thus, we preferred a dimensional perspective on syntactic complexity and diversity in psychiatric disorders. Cluster analysis showed four transdiagnostic clusters ranging from extremely complex to slightly complex speech that were accompanied by higher and lower FTD, respectively. Network analyses indicated differential networks across different clusters based on syntax. Notably, network associations between syntax, language-related neuropsychology, and psychopathology were more pronounced in higher syntax; cross-domain associations between syntax, language-related neuropsychology, and psychopathology were sparse in speech with lower syntax (i.e., slightly complex and moderately complex clusters).

Our results offer several new insights. First, using direct comparisons between HC, MDD, and SSD subjects, we were able to show numerous differences between the respective diagnostic categories with most pronounced distinctive features in SSD patients. These differences corroborate with previous studies^14,18,24,25,40,52,53^ on syntax. Moreover, we were able to extend these studies by using three further measures of syntactic complexity, allowing an in-depth investigation. Interestingly, all differences in these variables between groups were limited to SSD patients compared to HC and SSD compared to MDD, unlike no differences appeared between HC and MDD. However, significant differences across diagnostic categories indicated medium effect sizes (*η*^*2*^ ≥ 0.085). A larger sample size can lead to higher accuracy and reliability of the effect size.

Second, the multivariate pattern diagnostic classification showed weak classification rates, consequently the five variables of syntax itself represented no useful measures to classify patients regarding their clinical diagnosis. Early descriptions have shown syntactic complexity to be a marker separating SSD from mania, specifically in chronic courses^21,54–56^, whereas others showed a progressive reduction of syntactic complexity over time in SSD irrespective of the disease course^25^ but not in mania^57^. Taking a more transdiagnostic and dimensional view into account, we investigated sub-clusters of syntactic features across HC, MDD, and SSD. This approach corresponds to recent findings, showing a high overlap across different psychiatric disorders in several domains^1–6,32,58,59^ including behavioral and biological measures. This overlap is not considered when comparing or classifying clinical diagnoses^8–10^. In contrast, alternative (multivariate) methods are necessary to better understand psychiatric heterogeneity^6,32,60^. We found four transdiagnostic clusters expanding from extremely complex to slightly complex sentences. Interestingly, all identified clusters included HC, MDD, and SSD subjects with varying distributions (i.e., more HC and MDD in more complex clusters whereas the slightly complex cluster was mainly composed of SSD patients). Hence, different levels of syntax are also reflected by differences in language-related neuropsychology and psychopathology between clusters. Specifically the slightly complex cluster can be characterized by lowest language-related neuropsychological performance, pronounced negative symptoms, and higher amount of delusions in comparison to the other clusters. In contrast, participants of the extremely complex cluster exhibited less overall negative symptoms. These differences lead to the assumption that the identification of the clusters is related to the severity of psychopathological symptoms. The latter are in line with previous studies also highlighting the impact of negative symptoms on syntactic complexity^13,14,18,25,40,61,62^. Both subscales of SANS and SAPS for FTD are related with syntactic measures, yet they encompass a different scope of linguistic aspects. FTD is a broader concept in comparison to syntactic complexity, that focusses on a grammatical phenomenon of language^30^. Cognitive deficits such as impairment of language, memory, and executive functioning in consequence of negative symptoms are more strongly associated with difficulties in daily routines, social interaction, and resistance to therapy than positive symptoms^63–65^.

Third, network analyses were used to investigate the network-structure across the identified clusters. All networks showed high inter-relation and intra-relation within the parameters of the three different domains: syntax, language-related neuropsychology, and psychopathology. In all four cluster-based networks, measures of syntactic complexity were closely related while syntactic diversity appeared to be a separate node outside of the syntactic network. This emphasizes that syntactic complexity and diversity are, although related, two distinct concepts. Additionally, negative FTD and verbal episodic memory represented very relevant nodes in the networks. Both nodes mediate different domains. The connection between deficits in verbal episodic memory and SSD is well-known^66,67^. In reduced syntax (i.e., slightly and moderately complex clusters) cross-domain associations appeared to be very weak or missing. Furthermore, the direction of correlations varied within and across the clusters e.g., negative FTD was negatively correlated with pure syntactic complexity, positive FTD correlated positively with pure syntactic complexity in the extreme complex cluster, and the slightly complex cluster presented positive correlations for both. A negative relation between syntactic complexity and FTD is consistent with the literature; however, linguistic effects have been studied particularly in SSD and much less in MDD^11,30,68,69^. Negative FTD had a more influential function for disseminating information (high closeness centrality) than positive FTD. We assume an impact of different cluster size and common effect structures^70^. Future studies should investigate syntactic clusters and networks based on a larger sample size to explore stability and validity of our results. A beneficial extension would include the analysis of syntactic complexity and diversity in written language.

### Limitations

Some limitations must be noted. First, our sample size was relatively small, and the two clinical diagnoses were heterogeneous due to different disease severity. Nevertheless, speech performances were not associated with duration and severity of illness (i.e., number of hospitalizations, duration of hospitalization, and duration of current episode). Second, this study used a cross-sectional design which prohibits implications of causality. Third, education was significantly different between the extremely and the slightly complex clusters which might have influenced our results. Fourth, while some studies^15^ reported an impact of antipsychotic medication on syntactic complexity, others did not^25^. We did not find any medication effects. However, we cannot exclude potential effects of lifetime intake of psychiatric medication. Fifth, using a manual analysis of syntactic complexity and diversity instead of NLP algorithms entails some disadvantages such as lower comparability and lower efficiency. Nonetheless, an in-depth analysis was only achievable by using a manual approach.

## Conclusion

In conclusion, reduced syntactic complexity and diversity was mirrored in reduced performances in executive functioning, verbal fluency, and verbal episodic memory as well as in elevated positive and negative FTD. SSD produced significantly less complex sentences and significantly fewer, different types of complex sentences compared to MDD and HC. Clusters based on different degrees of syntax differed in language-related neuropsychological and psychopathological measures.

## Methods

### Participants

For the presented study we included *N* = 112 German-speaking participants (aged 20–67) who were part of the FOR2107 MACS cohort (data freeze of the October 20, 2022, for more details see Kircher et al., 2019, www.for2107.de). Patients were recruited from inpatient and outpatient facilities of the university hospital in Marburg and the departments of participating local hospitals within a 50 km radius of Marburg as well as via postings in local newspapers and flyers. The following exclusion criteria were applied: verbal IQ < 80, history of head trauma or unconsciousness, severe medical illnesses (cancer, autoimmune diseases, and infections), neurological illness, and the presence of a current substance dependence.

According to a semi-structured interview, including the Diagnostic and Statistical Manual of Mental Disorders, Fourth Edition (DSM-IV-TR)^71^, *n* = 34 participants were diagnosed with SSD, while *n* = 38 fulfilled the criteria for MDD. In addition, *n* = 40 individuals with no current or former history of any psychiatric disorder were included as HC. All procedures were approved by the local Ethics Committee according to the Declaration of Helsinki. Prior to study participation patients gave written informed consent and received a financial compensation. Table 1 shows an overview of descriptive statistics.

### Language-related neuropsychological assessment

We assessed the domains of executive functioning, VF and verbal episodic memory. VF was measured by using three different categories (60 seconds each): semantic VF (category “animals”), phonemic VF (initial letter “p”), and category alternating VF (alternating categories “sports” and “fruit”)^72^, determining semantic processing and executive functions. To test the performance of verbal episodic memory, we used the German version of the California Verbal Learning Test (VLMT)^73^.

### Psychopathological assessment

A number of psychopathological scales were assessed in the course of a semi-structured interview. Ratings were performed either during or following the interview. The level of global functioning was assessed with the Global Assessment of Functioning (GAF)^71^. The severity of MDD were measured by Hamilton Rating Scale for Depression (HAM-D)^74^ and Hamilton Anxiety Rating Scale (HAM-A)^75^. In addition, SANS^76^ and SAPS^77^ were administered, recording negative and positive symptoms in four subscales (see Table 1). Both SANS and SAPS include subscales for FTD that were very relevant for the following network analyses. All interviewers were familiar with and trained in the evaluation of the respective psychopathological scales. Interrater reliability was assessed with the interclass coefficient, achieving good reliability of *r* > 0.86 in all ratings and scales.

### Assessment of syntactic complexity and diversity

#### Eliciting speech

To elicit spontaneous speech, we used four pictures of the Thematic Apperception Test (TAT)^78^ which is in line to the procedures described by Liddle et al., 2002^79^. Instead of eight one-minute spontaneous speech samples, we assessed four different TAT pictures in three-minute periods; our aim was to elicit additional speech-related abbreviations (e.g., FTD) that potentially had not been present before the one-minute time frame but might develop over time. Participants were asked to tell a story about what might be happening in the picture. They were given a one-minute break between each picture; meanwhile the instruction was repeated and then the next picture was presented. If participants stopped within the three minutes of telling a story based on the picture, the instructor used non-directive prompts (e.g., “How do people feel?”; “What could happen next?”). Speech samples were audio recorded (Olympus WS-853) and transcribed literally using the f4transkript software (https://www.audiotranskription.de/f4transkript/). It is important to note that transcribers were unaware of the participants’ diagnoses.

### Analysis of transcripts

Transcripts were analyzed by total number of words (tokens), total number of different words (types), total number of sentences, mean length of utterance (MLU), type-token-ratio (TTR), simple sentences (main clauses without conjunctions or subordinations), coordinated sentences (sentences with conjunctions like “and/or” or enumerations without conjunctions) and 13 different types of complex sentences (main clause in combination with subordinate clause) (see Table 2) by KS. Complex sentences included 10 types of embedded adverbial clauses (temporal, local, modal, causal, conditional, adversative, final, consecutive, concessive and comparative), relative clauses, complement clauses and indirect questions. In contrast to Tavano et al., 2008, we excluded coordinated sentences from complex sentences, because simple sentences are often joined together by a conjunction, especially in oral speech production^14^. Thus, there is no embedded subordinate clause that is accompanied by a change in word order. For this reason, passive constructions were also neglected in our analysis. In addition to studies, that investigated syntactic complexity in SSD^25,40^, we intended to expand the knowledge with syntactic diversity inspired by Tavano et al., 2008 and extracted syntactic complexity and diversity as follows: The sum of all main clauses embedding subordinate clauses without overlaps over the total number of sentences resulted in a meaningful relative value for syntactic complexity (i.e., relative sum of subordinate clauses). Here, we did not distinguish between different depths of embedding. Thus, a complex sentence with only one embedded clause was on a par with a sentence that contained e.g., four embedded clauses. All complete utterances were assigned to either simple sentences, coordinated sentences, or complex sentences. However, overlaps between coordinated sentences and complex sentences could occur and were classified into both categories. The number of different types of complex sentences (0–13) that were produced in the picture description and divided by the maximum of possible different types (13) represents a relative value for syntactic diversity (i.e., syntactic diversity).

In addition to the metrics provided in Tavano et al., 2008, we calculated the following scores which allowed us a more detailed and comprehensive insight into syntactic complexity: 1. The sum of all subordinate clauses, as there are several of them in one main clause in relation to the number of all produced sentences (i.e., extended relative sum of subordinate clauses), but irrespective of various types of subordinate clauses in contrast to the third supplementary value (weighted sum of subordinate clauses). 2. The total number of subordinate clauses divided by the total number of complex sentences exclusively which allowed us to investigate syntactic complexity without confounding effects of non-complex sentences (i.e., pure syntactic complexity). 3. The total number of all subordinate clauses considering different types of complex sentences. The number of each subordinate clause multiplicated with a factor, which represents the number of different types in one main sentence (i.e., weighted sum of subordinate clauses), e.g., a main sentence contains 2 relative, 2 causal and 1 complement clause, implies a factor of 3 due to 3 types of complex sentences. Therefore, the value for this example is 15. For an overview of all analyzed categories, see Table 2.

### Statistical procedures

#### Group comparisons

Group differences in syntactic complexity and diversity between HC, MDD, and SSD groups were investigated with JASP (Version 0.16; JASP Team, 2021) using one-way ANOVA analyses. In case assumptions for parametric testing were not given, non-parametric Kruskal-Wallis test was used^80^. To investigate potential medication effects, we correlated the sum score of chlorpromazine equivalents^49^ (antipsychotics), Sackeim score^50^ (antidepressants), and the medication load index^51^ assessing both type and amount of different medication classes (antidepressants, antipsychotics, mood stabilizers) with the extracted syntactic complexity and diversity measures. Likewise, a correlation analysis was employed to test the relationship between duration and severity of illness (number of hospitalizations, duration of hospitalization, and duration of current episode), education, age and sex. All relevant information was obtained in the semi-structured interview and via self-reporting questionnaires.

### Classification

In the context of hypothesis testing, multivariate pattern classifications algorithm provides a mathematically solid framework to test if the combined data provides meaningful information about the variable of interest^81^. For the present analyses, we used five metrics related to syntactic complexity (see Tables 1, 2 for details on these measures) and support vector machines (SVM) with linear kernel^82^ to classify the data in three different combinations: HC from SSD, HC from MDD, and MDD from SSD. In each case, we used two-fold cross validation with 200 repetitions to estimate the classification accuracy. To estimate the statistical significance of accuracies, we used nonparametric permutation test during which we randomized the labels 1000 times per case and repeated the classification with permuted labels^83^. All classification analyses were performed using MATLAB R2021b.

### Cluster analysis

Cluster analysis was used to identify new sub-groups based on syntactic complexity and diversity. Therefore, we used the relative sum of subordinate clauses, extended relative sum of subordinate clauses, pure syntactic complexity, weighted sum of subordinate clauses, and syntactic diversity. The random forest algorithm implemented in JASP was used to identify clusters of participants that performed similarly in terms of syntax irrespective of a diagnosis. The random forest algorithm bases on several tree predictors that contrast the similarities and dissimilarities of measurements^84^. We did not fix a number of clusters beforehand; instead, we determined optimized clusters according to the BIC. MANCOVA interaction analyses were conducted to test if clinical diagnoses affected cluster contribution. An interaction effect was investigated for all five values for syntactic complexity and diversity. Moreover, we compared extracted clusters with regard to the above medication, duration and severity of illness, education, age, and sex variables.

Next, we compared language-related neuropsychological and psychopathological data between obtained clusters to better characterize them using one-way ANOVAs or Kruskal-Wallis tests^80^ (see Extended Data Table 3).

#### Network analyses of syntax, language-related neuropsychology and psychopathology

Further, network analyses based on the Gaussian Graphical Model (GGM) were used to investigate the relationship between multiple variables of syntax, language-related neuropsychology, and psychopathology in delineated clusters^85^. Based on the literature^14,34,35,37,86^, eleven language-related variables, i.e., five variables of syntactic complexity, four language-related neuropsychological and two psychopathological variables were chosen and networks were calculated for each cluster separately (from previous cluster analysis) and additionally for the total sample (see Fig. 1), both with 1000 permutations for non-parametric bootstrapping. All variables are visualized as nodes and significant correlations between two variables as edges. The thickness and intensity of color of the edges indicate the strength of correlations; blue edges mark positive correlations and red edges mark negative correlations. The Extended Bayesian Information Criterion (EBIC)^87^ Graphical Least Absolute Shrinkage and Selection Operator (Lasso)^88^ (short EBICglasso) was used for estimation, with tuning parameter of 0.25. Partial correlations between the selected parameter were estimated and small edges were reduced to zero^70,89^. As opposed to a non-regularized model^70^, this method leads to sparser networks with missing connections between the nodes^90^. It should be noted that missing edges are the least important and non-existing edges will not be presented in a network based on GGM^70^. Four centrality measures indicate different relations of nodes: 1. betweenness (i.e., how many times a node is on the shortest path between two nodes), 2. closeness (i.e., how close is a node to other nodes), 3. strength (i.e., sum of connections irrespective of negative or positive) and 4. expected influence (i.e., sum of connections, accounts for negative and positive correlations)^90^. The Fruchterman-Reingold algorithm was the basis of the layout of our networks^91^. This algorithm led to a weighted positioning of nodes in the network^91^. We used JASP to perform the network analyses on the basis of bootnet^90^ and the qgraph packages^92^ in R to create the graphs.

## Supplementary information

Extended Data Table 1

Extended Data Table 2

Extended Data Table 3

Extended Data Table 4

Extended Data Figure 1

Extended Data Figure 2

Extended Data Tables and Figure legend

## Data Availability

The data and code supporting the findings of this study can be accessed by contacting the corresponding author (KS).

## Code availability

The data and code supporting the findings of this study can be accessed by contacting the corresponding author (KS).
