# Supplementary material for: Syntactic complexity and diversity of spontaneous speech production in schizophrenia spectrum and major depressive disorders
Source: Schizophrenia (Heidelb). 2023 May 29;9(1):35. doi: 10.1038/s41537-023-00359-8 (PMC10227047; doi:10.1038/s41537-023-00359-8)
Supplement: Supplementary file 1 — Extended Data Table 1 [file 41537_2023_359_MOESM1_ESM.docx]

**Extended Data Table 1. Correlations of syntax with education, age, sex, number of hospitalizations, duration of hospitalization, and duration of current episode**

|  | Education | Age | Sex | Number of hospitalizations | Duration of hospitali-zation | Duration of current episode |
| --- | --- | --- | --- | --- | --- | --- |
| **Syntax** |  |  |  |  |  |  |
| relative sum of subordinate clauses | *r*=.199; *p*=.040 | *r*=­-.139; *p*=.144 | *r*=.097; *p*=.308 | *r*=-.141; *p*=.140 | *r*=-.083; *p*=.385 | *r*=-.003; *p*=.988 |
| extended relative sum of subordinate clauses | *r*=.253; *p*=.009 | *r*=-.135; *p*=.156 | *r*=.113; *p*=.237 | *r*=-.133; *p*=.163 | *r*=-.091; *p*=.340 | *r*=.021; *p*=.903 |
| pure syntactic complexity | *r*=.236; *p*=.015 | *r*=-.061; *p*=.520 | *r*=.120; *p*=.207 | *r*=-.123; *p*=.200 | *r*=-.107; *p*=.262 | *r*=-.032; *p*=.855 |
| weighted sum of subordinate clauses | *r*=.250; *p*=.010 | *r*=-.152; *p*=.110 | *r*=.090; *p*=.347 | *r*=-.132; *p*=.166 | *r*=-.121; *p*=.205 | *r*=-.013; *p*=.939 |
| syntactic diversity | ***r*=.333; *p*<.001** | *r*=-.155; *p*=.104 | *r*=.026; *p*=.787 | *r*=-.102; *p*=.286 | *r*=-.205; *p*=.031 | *r*=-.168; *p*=.333 |

Bold font indicates significant results after correcting for multiple testing (Bonferroni).
