# Supplementary material for: Syntactic complexity and diversity of spontaneous speech production in schizophrenia spectrum and major depressive disorders
Source: Schizophrenia (Heidelb). 2023 May 29;9(1):35. doi: 10.1038/s41537-023-00359-8 (PMC10227047; doi:10.1038/s41537-023-00359-8)
Supplement: Supplementary file 2 — Extended Data Table 2 [file 41537_2023_359_MOESM2_ESM.docx]

**Extended Data Table 2.** Cluster comparisons in education, age, sex, number of hospitalizations, duration of hospitalization, and duration of current episode

|  | Education | Age | Sex | Number of hospitali-zations | Duration of hospitali-zation | Duration of current episode |
| --- | --- | --- | --- | --- | --- | --- |
| **Cluster comparisons** |  |  |  |  |  |  |
| Extremely – very | *d*=-.473; *p*=.528 | *d*=.310; *p*=.815 | *V*=.230; *p*=.152 | *d*=.314; *p*=.903 | *d*=.293; *p*=.868 | *d*=-.658; *p*=.797 |
| Extremely – moderately | *d*=-.776; *p*=.044 | *d*=.217; *p*=.846 | *V*=.017; *p*=.898 | *d*=.283; *p*=.709 | *d*=.164; *p*=.931 | *d*=-.203; *p*=.977 |
| Extremely – slightly | *d*=1.025; ***p*=.005** | *d*=-.251; *p*=.808 | *V*=.230; *p*=.091 | *d*=-.486; *p*=.437 | *d*=-.426; *p*=.552 | *d*=.239; *p*=.958 |
| Very – moderately | *d*=-.287; *p*=.731 | *d*=-.057; *p*=.997 | *V*=.234; *p*=.075 | *d*=.069; *p*=.993 | *d*=-.073; *p*=.992 | *d*=.415; *p*=.903 |
| Very –  slightly | *d*=.518; *p*=.259 | *d*=.032; *p*=1.00 | *V*=.009; *p*=.948 | *d*=-.219; *p*=.888 | *d*=-.130; *p*=.971 | *d*=-.336; *p*=.932 |
| Moderately – slightly | *d*=.241; *p*=.735 | *d*=-.026; *p*=.999 | *V*=.255; *p*=.029 | *d*=-.118; *p*=.943 | *d*=-.194; *p*=.815 | *d*=.047; *p*=.999 |

Bold font indicates significant results after correcting for multiple testing (Bonferroni).
