# Supplementary material for: Syntactic complexity and diversity of spontaneous speech production in schizophrenia spectrum and major depressive disorders
Source: Schizophrenia (Heidelb). 2023 May 29;9(1):35. doi: 10.1038/s41537-023-00359-8 (PMC10227047; doi:10.1038/s41537-023-00359-8)
Supplement: Supplementary file 3 — Extended Data Table 3 [file 41537_2023_359_MOESM3_ESM.docx]

**Extended Data Table 3.** Cluster syntactic complexity and diversity

|  | Extremely complex cluster  (n=20) | Very complex cluster  (n=19) | Moderately complex cluster (n=39) | Slightly complex cluster  (n=34) | Group comparison | Effect size |
| --- | --- | --- | --- | --- | --- | --- |
| **Syntax** |  |  |  |  |  |  |
| relative sum of subordinate clauses | .57 (.08) | .42 (.04) | .40 (.09) | .26 (.06) | ***p*= <.001^a^**  (F=76.84) | *η^2^*=.681 |
| extended relative sum of subordinate clauses | 1.12 (.26) | .75 (.05) | .57 (.09) | .34 (.09) | ***p=* .000^b^**  (H=98.92) | *η^2^*=.810 |
| pure syntactic complexity | 1.97 (.28) | 1.82 (.14) | 1.47 (.19) | 1.29 (.14) | ***p=* <.001^b^**  (H=75.39) | *η^2^*=.663 |
| weighted sum of subordinate clauses | 2.24 (.84) | 1.40 (.15) | .88 (.20) | .46 (.14) | ***p=* .000^b^**  (H=98.97) | *η^2^*=.733 |
| syntactic diversity | .72 (.14) | .65 (.12) | .58 (.11) | .51 (.11) | ***p=* <.001^c^**  (F=15.89) | *η^2^*=.306 |
| **Neuropsychology** |  |  |  |  |  |  |
| semantic VF | 22.58 (5.46) | 21.22 (5.01) | 23.60 (4.6) | 19.42 (5.82) | ***p=* .012^d^**  (F=3.84) | *η^2^*=.102 |
| phonemic VF | 11.74 (4.46) | 9.33 (3.46) | 11.92 (4.32) | 9.23 (3.75) | *p=* .016^d^  (F=3.59) | *η^2^*=.096 |
| alternating VF | 15.37 (3.04) | 14.44 (3.01) | 15.03 (2.59) | 11.97 (4.14) | ***p=* <.001^e^**  (F=6.42) | *η^2^*=.160 |
| verbal episodic memory^1^ | 56.58 (9.48) | 55.33 (13.04) | 59.03 (8.17) | 49.97 (10.53) | ***p=* .004^d^**  (F=4.75) | *η^2^*=.123 |
| **Psychopathology** |  |  |  |  |  |  |
| GAF | 75.00 (16.68) | 73.46 (22.12) | 75.44 (17.81) | 60.08 (19.30) | ***p=* .003^e^**  (F=4.88) | *η^2^*=.119 |
| HAM-D 17 | 3.99 (6.64) | 3.46 (4.46) | 4.64 (6.04) | 6.22 (7.16) | *p=* .405  (F=.98) | *η^2^*=.027 |
| HAM-A | 7.25 (9.31) | 6.43 (6.75) | 6.07 (7.94) | 8.34 (9.26) | *p=* .699  (F=.48) | *η^2^*=.013 |
| SANS sum | 3.93 (9.77) | 4.84 (5.79) | 6.33 (9.71) | 12.26 (13.12) | *p=* .005^f^  (H=12.76) | *η^2^*=.094 |
| SANS affect | 1.11 (3.41) | 2.02 (3.93) | 1.98 (3.65) | 4.09 (4.94) | *p=* .008^f^  (H=11.87) | *η^2^*=.071 |
| SANS alogia^2^ | .50 (1.32) | .75 (1.31) | 1.21 (2.14) | 1.37 (2.12) | *p=* .338  (F=1.14) | *η^2^*=.031 |
| SANS avolition | .70 (1.66) | 1.42 (1.79) | 1.57 (2.96) | 3.80 (4.00) | ***p=*.001^g^**  (H=16.12) | *η^2^*=.140 |
| SANS anhedonia | 1.60 (3.66) | 1.18 (1.92) | 1.52 (3.18) | 3.02 (4.67) | *p=* .215  (F=1.51) | *η^2^*=.040 |
| SAPS sum | .81 (1.76) | 4.21 (9.08) | 3.68 (7.43) | 9.31 (14.03) | *p=* .166  (H=5.08) | *η^2^*=.095 |
| SAPS hallucinations | .10 (.31) | .42 (1.12) | .51 (2.75) | 1.86 (4.30) | *p=* .024^h^  (H=9.45) | *η^2^*=.055 |
| SAPS delusions | .00 (.00) | 1.05 (2.80) | 1.03 (2.75) | 3.34 (5.82) | *p=* .006^f^  (H=12.39) | *η^2^*=.101 |
| SAPS bizarre behaviour | .05 (.22) | .26 (.65) | .05 (.22) | .55 (.96) | *p=* .011^h^  (H=11.12) | *η^2^*=.116 |
| SAPS positive FTD | .65 (1.60) | 2.81 (6.13) | 2.08 (3.89) | 4.30 (7.58) | *p=* .225  (H=4.36) | *η^2^*=.055 |

Means and standard deviations (SD) (in brackets) are listed for each group and category. Pairwise comparisons: ^a^= all clusters significant besides moderately complex and very complex. ^b^= all clusters significant besides very complex and extremely complex. ^c^= slightly complex < very complex, extremely complex; moderately complex < extremely complex. ^d^= slightly complex < moderately complex. ^e^= slightly complex < moderately complex, extremely complex. ^f^= slightly complex > extremely complex. ^g^= slightly complex > moderately complex, extremely complex. ^h^= slightly complex > moderately complex.

Bold font indicates significant results after correcting for multiple testing (Bonferroni).

^1^ Sum of correct words. ^2^ Subscale used for negative FTD.
