# Supplementary material for: Syntactic complexity and diversity of spontaneous speech production in schizophrenia spectrum and major depressive disorders
Source: Schizophrenia (Heidelb). 2023 May 29;9(1):35. doi: 10.1038/s41537-023-00359-8 (PMC10227047; doi:10.1038/s41537-023-00359-8)
Supplement: Supplementary file 4 — Extended Data Table 4 [file 41537_2023_359_MOESM4_ESM.docx]

**Extended Data Table 4.** Centrality measures of networks

|  | All  (n=112) | | | | Extremely complex cluster  (n=20) | | | | Very complex cluster  (n=19) | | | | | Moderately complex cluster (n=39) | | | | | Slightly complex cluster  (n=34) | | | | |
| --- | --- | --- | --- | --- | --- | --- | --- | --- | --- | --- | --- | --- | --- | --- | --- | --- | --- | --- | --- | --- | --- | --- | --- |
| **Non-zero edges**  **Sparsity index** | 34/55  .38 | | | | 48/55  .13 | | | | 41/55  .26 | | | | | 12/55  .78 | | | | | 33/55  .40 | | | | |
|  | B | C | S | EI | B | C | S | EI | B | C | S | EI | B | | C | S | EI | B | | C | S | EI |  |
| **Syntax** |  |  |  |  |  |  |  |  |  |  |  |  |  | |  |  |  |  | |  |  |  |  |
| relative sum of subordinate clauses | -.86 | -.95 | .87 | -.75 | -.50 | -.84 | -.26 | -.31 | -.87 | -1.10 | .24 | -.91 | -.21 | | .00 | .98 | -.88 | .37 | | .40 | 1.32 | -.68 |  |
| extended relative sum of subordinate clauses | .92 | -.22 | 2.25 | 2.29 | 1.01 | -.21 | .29 | 1.91 | 2.33 | -.26 | 1.82 | 1.67 | -.21 | | .00 | 1.46 | 1.89 | -.42 | | -.37 | 1.17 | 1.78 |  |
| pure syntactic complexity | -.86 | -1.64 | .85 | -.69 | -.50 | -.74 | -.03 | -.66 | -.87 | -1.07 | .71 | -1.03 | -1.00 | | .00 | .62 | -1.20 | .11 | | 1.06 | 1.08 | .32 |  |
| weighted sum of subordinate clauses | 1.51 | .36 | -.03 | .85 | 1.20 | 1.30 | -.94 | .39 | .62 | .31 | -.32 | .24 | -1.00 | | .00 | 1.09 | 1.57 | -.68 | | -.39 | .18 | 1.10 |  |
| syntactic diversity | 1.86 | 1.97 | -1.25 | -.83 | -.87 | -1.74 | -1.82 | -.55 | -.87 | .50 | -.57 | -1.00 | -1.00 | | .00 | -1.89 | -1.01 | -.68 | | -1.78 | -1.63 | -1.26 |  |
| **Neuropsychology** |  |  |  |  |  |  |  |  |  |  |  |  |  | |  |  |  |  | |  |  |  |  |
| semantic VF | -.63 | -.52 | -.06 | .57 | -.87 | .02 | .46 | -.48 | -.02 | .67 | .31 | .35 | .57 | | .00 | -.40 | .13 | -.68 | | -.33 | -.65 | .20 |  |
| phonemic VF | -.86 | -.70 | -.73 | .28 | -.87 | -.43 | -.84 | -.40 | -.87 | -2.09 | -2.24 | -1.06 | .57 | | .00 | -.52 | .17 | -.68 | | -.79 | -.82 | .42 |  |
| alternating VF | .32 | 1.12 | .04 | .48 | -.68 | .20 | .75 | -.98 | -.45 | .55 | -.13 | 1.71 | .57 | | .00 | -.71 | -.49 | 1.82 | | 1.03 | .94 | .82 |  |
| verbal episodic memory^1^ | -.39 | .12 | -.37 | -.59 | -.68 | -.08 | .50 | 1.66 | .41 | .84 | .05 | .18 | .57 | | .00 | -.79 | -.49 | -.42 | | -.40 | -.79 | -.73 |  |
| **Psychopathology** |  |  |  |  |  |  |  |  |  |  |  |  |  | |  |  |  |  | |  |  |  |  |
| negative FTD | -.39 | .55 | -.66 | -.71 | 1.39 | 1.81 | -.07 | .44 | .84 | .60 | .59 | -.25 | -1.00 | | .00 | -.23 | .42 | 1.96 | | 1.81 | -.23 | -1.23 |  |
| positive FTD | -.63 | -.09 | -.90 | -.90 | 1.39 | .72 | 1.98 | -1.04 | -.23 | 1.03 | -.47 | .11 | 2.14 | | .00 | .39 | -.12 | -.68 | | -.25 | -.58 | -.74 |  |

Betweenness (B), closeness (C), strength (S) and expected influence (EI) are the four centrality measures for all variables of network analyses (see figure 1).

^1^ Values are the total sum of correct words.
