# Supplementary figures and images for: Syntactic complexity and diversity of spontaneous speech production in schizophrenia spectrum and major depressive disorders
Source: Schizophrenia (Heidelb). 2023 May 29;9(1):35. doi: 10.1038/s41537-023-00359-8 (PMC10227047; doi:10.1038/s41537-023-00359-8)

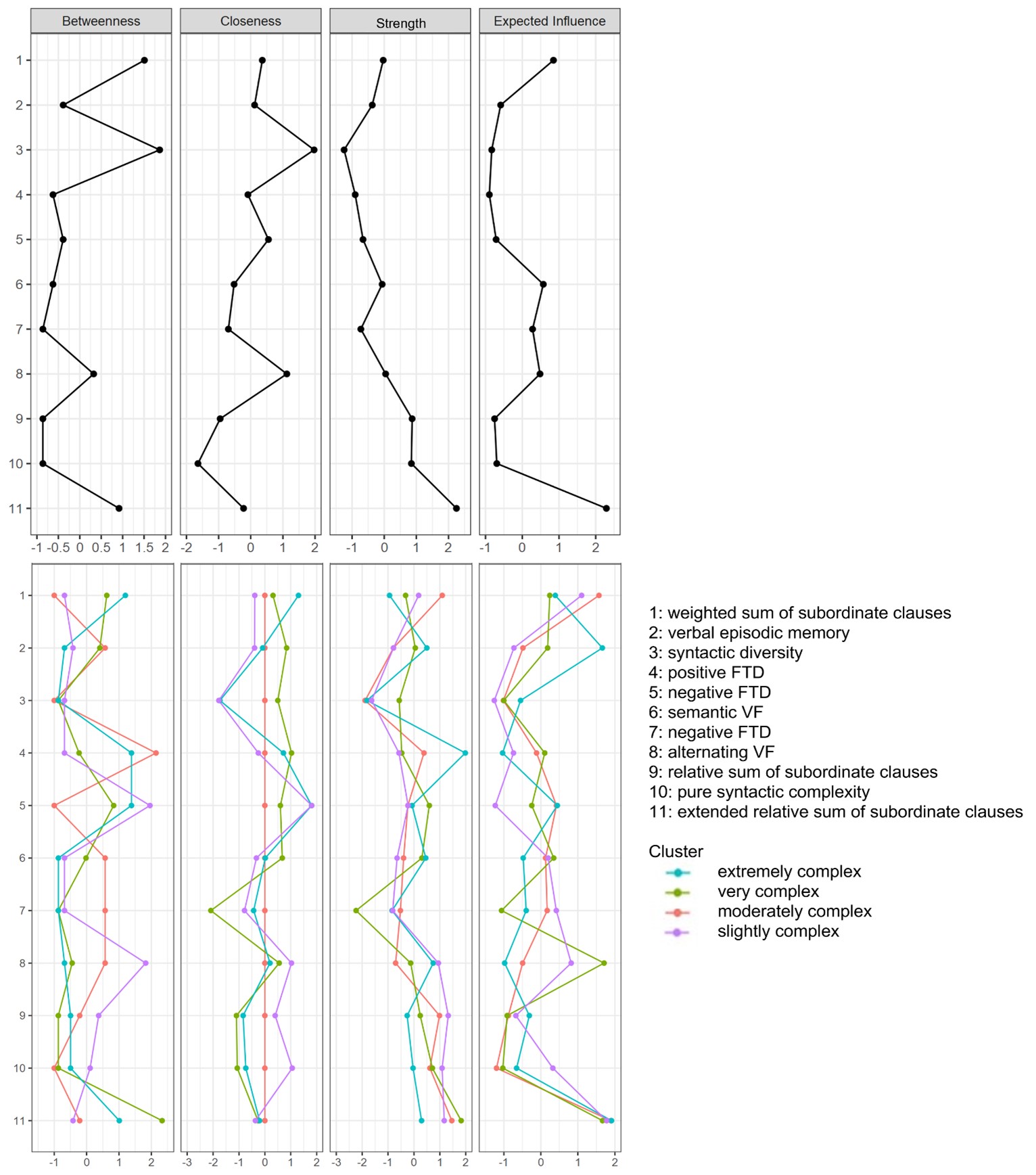

Supplement: Supplementary file 5 — Extended Data Figure 1 [file 41537_2023_359_MOESM5_ESM.jpg]

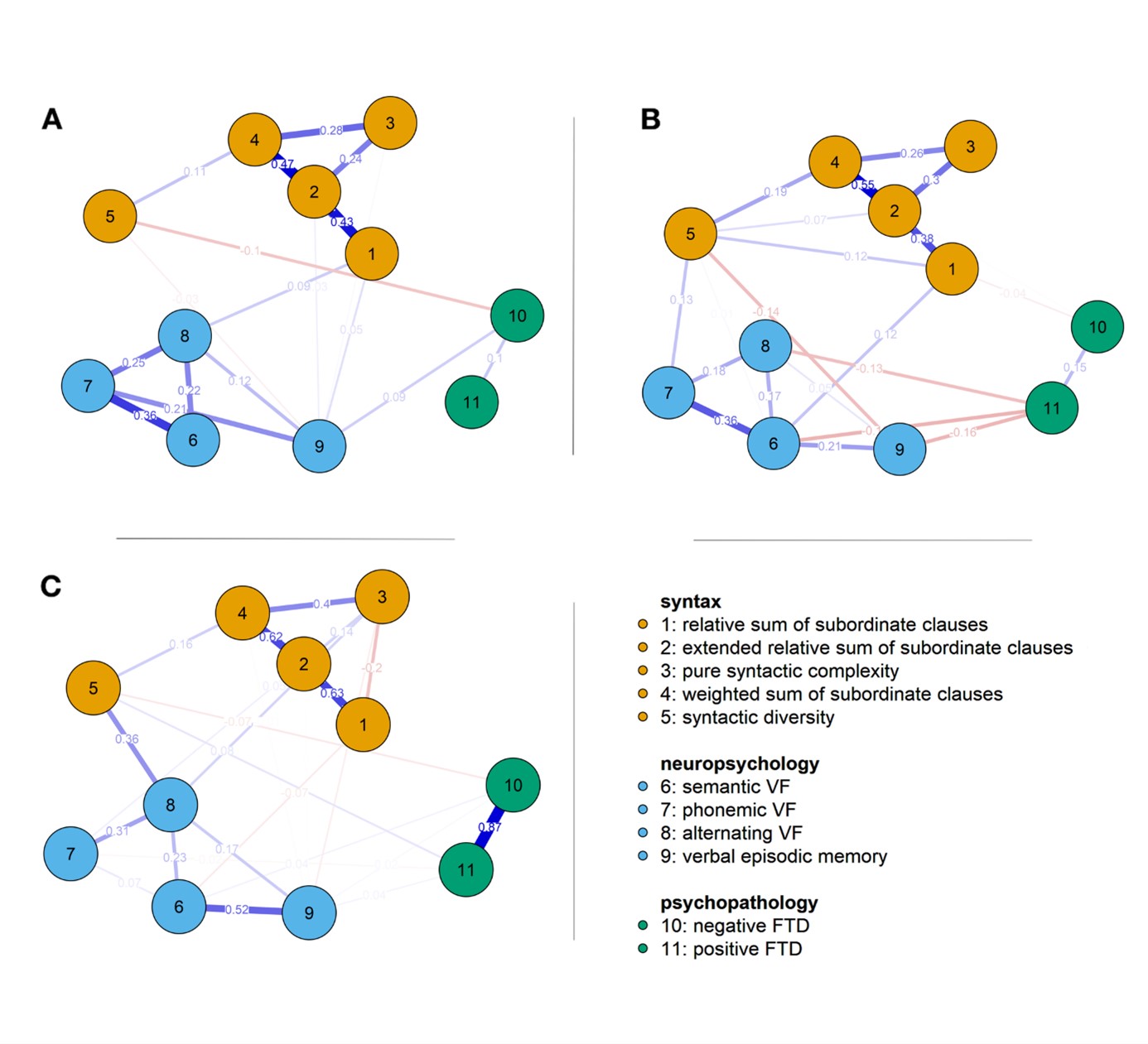

Supplement: Supplementary file 6 — Extended Data Figure 2 [file 41537_2023_359_MOESM6_ESM.jpg]
