# Supplementary material for: Syntactic complexity and diversity of spontaneous speech production in schizophrenia spectrum and major depressive disorders
Source: Schizophrenia (Heidelb). 2023 May 29;9(1):35. doi: 10.1038/s41537-023-00359-8 (PMC10227047; doi:10.1038/s41537-023-00359-8)
Supplement: Supplementary file 7 — Extended Data Tables and Figure legend [file 41537_2023_359_MOESM7_ESM.docx]

**Extended Data Table 1. Correlations of syntax with education, age, sex, number of hospitalizations, duration of hospitalization, and duration of current episode**

Bold font indicates significant results after correcting for multiple testing (Bonferroni).

**Extended Data Table 2.** Cluster comparisons in education, age, sex, number of hospitalizations, duration of hospitalization, and duration of current episode

Bold font indicates significant results after correcting for multiple testing (Bonferroni).

**Extended Data Table 3.** Cluster syntactic complexity and diversity

Means and standard deviations (SD) (in brackets) are listed for each group and category. Pairwise comparisons: ^a^= all clusters significant besides moderately complex and very complex. ^b^= all clusters significant besides very complex and extremely complex. ^c^= slightly complex < very complex, extremely complex; moderately complex < extremely complex. ^d^= slightly complex < moderately complex. ^e^= slightly complex < moderately complex, extremely complex. ^f^= slightly complex > extremely complex. ^g^= slightly complex > moderately complex, extremely complex. ^h^= slightly complex > moderately complex.

Bold font indicates significant results after correcting for multiple testing (Bonferroni).

^1^ Sum of correct words. ^2^ Subscale used for negative FTD.

**Extended Data Table 4.** Centrality measures of networks

Betweenness (B), closeness (C), strength (S) and expected influence (EI) are the four centrality measures for all variables of network analyses (see figure 1).

^1^ Values are the total sum of correct words.

**Extended Data** **Figure 1** **Centrality plot of network measures.** They are listed in Extended Data table 4. The plot above refers to all participants and below to clusters.

**Extended Data** **Figure 2** **Networks of SSD, MDD and HC.** Networks using the EBICglasso method in groups: SSD (A), MDD (B) and HC (C) based on Gaussian Graphical Model including variables of syntax, neuropsychology, and psychopathology. All correlations illustrated in the networks as edges are regularized partial correlations and stronger than 0.1 and -0.1. Estimated correlations with the value 0 are not visualized in the network. Orange nodes are part of syntax represented by (1) relative sum of subordinate clauses; (2) extended relative sum of subordinate clauses; (3) pure syntactic complexity; (4) weighted sum of subordinate clauses; (5) syntactic diversity, blue nodes to neuropsychology represented by (6) semantic VF; (7) lexical VF; (8) alternating VF; (9) verbal episodic memory, and green nodes to psychopathology represented by (10) negative FTD and (11) positive FTD. Blue connections indicate positive relationships, red connections mark negative relationships. The thickness of lines represents the weight of connection.
